# Contrasting response of coexisting plant’s water-use patterns to experimental precipitation manipulation in an alpine grassland community of Qinghai Lake watershed, China

**DOI:** 10.1371/journal.pone.0194242

**Published:** 2018-04-20

**Authors:** Huawu Wu, Jing Li, Xiao-Yan Li, Bin He, Jinzhao Liu, Zhiyun Jiang, Cicheng Zhang

**Affiliations:** 1 Key Laboratory of Watershed Geographic Sciences, Nanjing Institute of Geography and Limnology Chinese Academy of Sciences, Nanjing, China; 2 Nanjing Institute of Geography and Limnology Chinese Academy of Sciences, Nanjing, China; 3 Key Laboratory of Grassland Ecosystem (Gansu Agricultural University), Ministry of Education, Gansu, China; 4 Tourism School, Jiujiang College, Jiujiang, China; 5 State Key Laboratory of Earth Surface Processes and Resource Ecology, Beijing Normal University, Beijing, China; 6 School of Natural Resources, Faculty of Geographical Science, Beijing Normal University, Beijing, China; 7 State key Laboratory of Loess and Quaternary Geology, Institute of Earth Environment, Chinese Academy of Sciences, Xi’an, China; 8 School of Geography, South China Normal University, Guangzhou, China; Estacion Experimental del Zaidin, SPAIN

## Abstract

Understanding species-specific changes in water-use patterns under recent climate scenarios is necessary to predict accurately the responses of seasonally dry ecosystems to future climate. In this study, we conducted a precipitation manipulation experiment to investigate the changes in water-use patterns of two coexisting species (*Achnatherum splendens* and *Allium tanguticum*) to alterations in soil water content (SWC) resulting from increased and decreased rainfall treatments. The results showed that the leaf water potential (Ψ) of *A*. *splendens* and *A*. *tanguticum* responded to changes in shallow and middle SWC at both the control and treatment plots. However, *A*. *splendens* proportionally extracted water from the shallow soil layer (0–10cm) when it was available but shifted to absorbing deep soil water (30–60 cm) during drought. By contrast, the *A*. *tanguticum* did not differ significantly in uptake depth between treatment and control plots but entirely depended on water from shallow soil layers. The flexible water-use patterns of *A*.*splendens* may be a key factor facilitating its dominance and it better acclimates the recent climate change in the alpine grassland community around Qinghai Lake.

## Introduction

Water, disturbance, and edaphic factors determine the species distribution, composition, and productivity of arid and semiarid regions [1–3]. Precipitation in these regions is characterized by high temporal and spatial variability, affecting the amount of soil water available to plants [4]; thus, plants might be expected to respond rapidly to obtain water from different soil depths. However, the frequency of drought periods and the precipitation intensity is projected to increase over the coming decades in northwest regions of China, such as Qinghai, and the eastern regions of Gansu and Inner Mongolia, as a result of climate change [5, 6]. Moreover, results from regional models (e.g., RegCM4) have shown that the spring and summer seasons in these regions are subject to an increased frequency of drought [7], impacting the productivity, distribution, and species compositions of alpine grassland communities in eastern regions of the Tibetan Plateau [5].

Scenarios predict that mean precipitation is also projected to increase at middle and high latitude regions, and both increases and decreases in consecutive dry days between precipitation events are observed in these regions [1, 8]. These alterations of precipitation induce the changes of water status of terrestrial ecosystem takes great impacts on net primary productivity (NPP), species diversity, and plant community structure [9]. Previous studies indicate that long-term water addition in grasslands either increased [10] or had little consistent effect on species diversity [11], which are related with differential plants water uptake among associated species [12, 13]. Flexible water uptake patterns by plant facilitate its competitions among different plant functional types in the water-limited habitats [14, 15]. Hence, detecting the effects of altered precipitation in the water-limited ecosystems requires understanding how species will respond to drought or increases in precipitation.

Although the ecological effects of spring and summer droughts on grasslands have been investigated in different rainfall manipulation experiments [2, 3], most of these field studies have focused on either C_4_-grass dominated prairies in arid North America [16] or C_3_-grasslands in Europe [17], whereas a limited number of studies have concentrated on alpine grasslands that are widely distributed in the eastern regions of the Tibetan Plateau. In general, previous studies have shown that, when plants in a water-limited environment were exposed to drought, their root growth was enhanced more quickly than their aboveground shoot growth, resulting in more carbon being transported to belowground organs [18–20]. The results from these studies conform with a fundamental prediction of plant ecology: that is, that plant roots will extend into deeper soil layers to capture enough water, thus avoiding the low levels of water contained at shallower soil depths as a result of drought [21, 22]. However, it remains uncertain whether these alterations in root distribution and density result in plants using water from the deeper soil layers. For example, some authors have pointed out that the distribution of roots is not necessarily correlated with the depth of soil water absorption [23, 24]. Hence, the question of whether drought results in a shift to stable soil water sources in alpine grasslands remains unresolved.

To fill the aforementioned knowledge gaps, we investigated the water-use patterns of coexisting plant species in an alpine grassland community and their responses to changes in soil water availability under precipitation manipulation experiments during two consecutive growing seasons in 2013 and 2014, in Qinghai Lake watershed, China. In this study site, the *Achnatherum splendens* grassland community is dominated by *A*. *splendens*, *Leymus chinensis*, *Heteropappus altaicus*, and *Allium tanguticum* around Qinghai Lake, but its species distribution and richness are affected by changes in precipitation, such as a long run of consecutive dry days and extreme precipitation events [25, 26]. However, the effects of drought and irrigation treatment (e.g., prolonged drought or intensified precipitation events) have, in general, become unconspicuous over time [12]. The characterization of seasonal changes in the water sources of plants is crucial for understanding the mechanisms underlying these species-specific responses to variability in soil water availability. Moreover, extreme drought and precipitation events during the study period enabled us to clearly identify the species-specific differences in the use of water sources, and to accurately predict community shifts in response to future climate. Thus, the current study addressed the following questions: (1) What are the water sources for each plant species and do they change over time? (2) How do the water-use patterns of coexisting species respond to drought and irrigation treatments?

## Materials and methods

### Study site description

**Fig 1:**
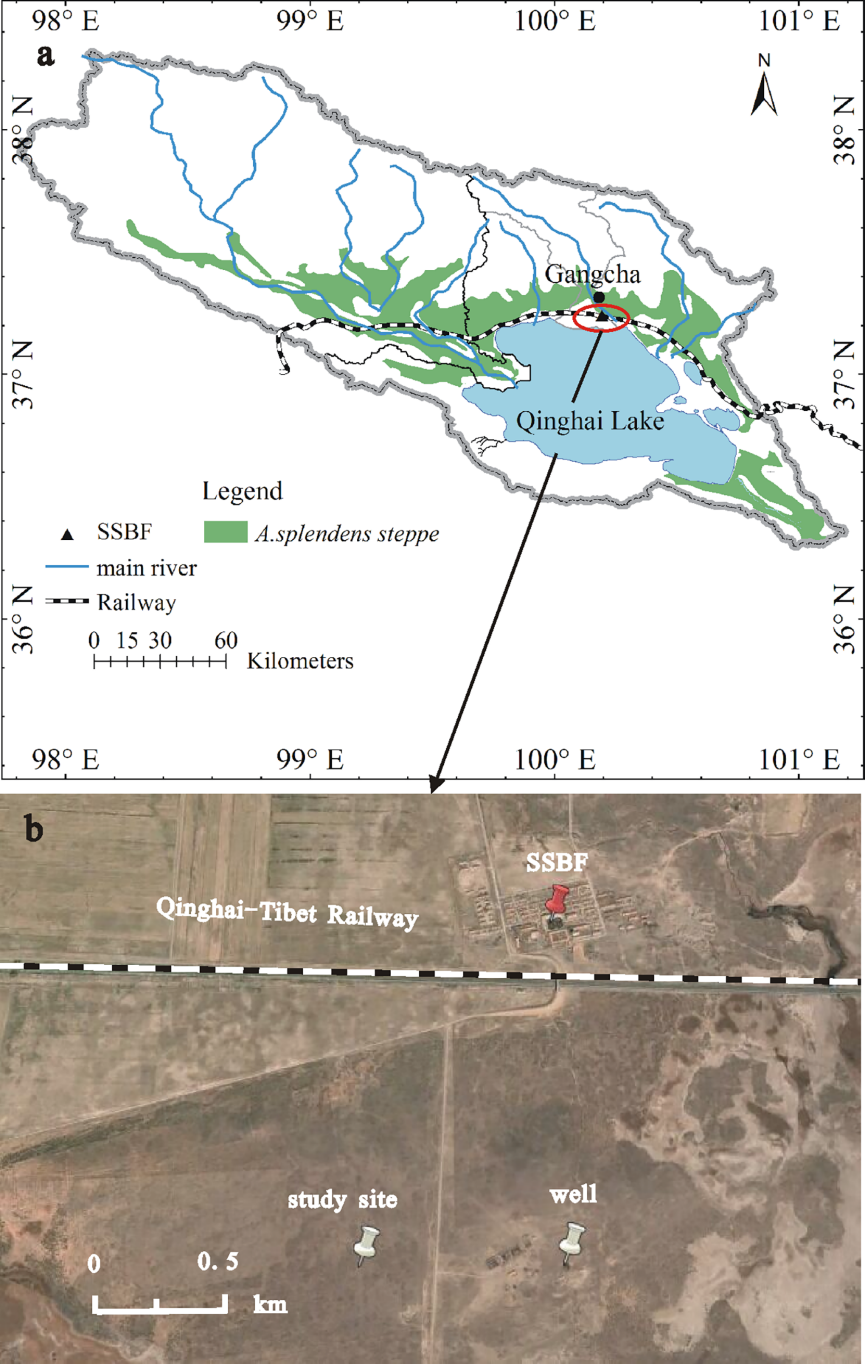
The geographic location of the Qinghai Lake watershed on the Qinghai-Tibet Plateau (a) and the study site (b, SSBF: Sanjiaocheng Sheep Breeding Farm).

The Qinghai Lake watershed is a hydrologically closed, high-elevation, intermountain watershed on the northeastern Tibetan Plateau (Fig 1A). It is situated in a semiarid, cold, alpine climate zone that is exposed to the East Asian monsoon during the growing season. The mean annual temperature of the drainage basin is 0.1°C, and the mean annual precipitation is approximately 400 mm, more than 65% of which falls between June and September. The mean annual evaporation is approximately 1300 mm, over 65% of which occurs during the summer [27, 28]. The growing season is from early June to late September.

The study site is located to the north of Qinghai Lake (Fig 1B). The *A*. *splendens* grassland community is mainly distributed around Qinghai Lake (Fig 1A) and has vital roles in the conservation of both water and soil [29]. The most suitable habitats for this grass species are lacustrine plains, valley terraces, and piedmont ramps. Erect livestock fences have been in place since the 1980s to protect the *A*. *splendens* grassland, which is used as a winter and spring grazing pasture for yak and sheep from February to June. The community structure is characterized by two distinct vertical stratifications: an overstory (comprising deep-rooted tussock tallgrass, *A*. *splendens*) and an understory (comprising other shallow-rooted species). The understory layer is dominated by perennial and shallow-rooted species of mixed herbs, such as *Agropyron cristatum*, *Carex ivanovae*, *H*. *altaicus*, and *A*. *tanguticum*, and by grasses, such as *Stipa breviflora* and *L*. *chinensis*, which enclose *A*. *splendens* tussocks [30]. The soil depth in study site was approximately 60–100 cm and had a silty loam texture.

### Experimental design

The experiment was conducted over two consecutive years (2013 and 2014) and comprised six plots (each 2×2.8 m) in the study site. Three plots (randomly selected) were subject to drought and irrigation treatments, whereas the other three plots were control plots. Each treatment plot was covered with a 2.4m ×3 m transparent rainout shelter during the growing season (S1 Fig, Table 1). During each drought treatment period, the excluded precipitation amount: total precipitation amount ratio was 18.8% and 54.3% in 2013 and 2014, respectively (Table 1). Shelter roofs were approximately 0.9–1 m above the ground, and open on all four sides to maximize air movement and minimize artifacts resulting from temperature and relative humidity. The duration of the rainout period was set to create drought conditions during the peak growing season by reducing the total amount of precipitation during the growing season (from June to August) in 2013 and 2014 (Table 1). Additionally, to avoid lateral soil water flow, a trench (approximately 60 cm deep) was excavated around the rainout plots and covered with a waterproof plastic film.

**Table 1:** Overview of the air temperature, precipitation amount between July and August, treatment period (sheltered installed) and excluded precipitation amount for two consecutive years.

Year	T (°C)	P (mm)	Treatment periods		Excluded precipitation amount(mm)^a^
			drought	irrigation	
2013	12.6	306.8	5 July-18 July	18 July-1 August	57.7(18.8%)
2014	10.3	313.4	5 July-1 August	1 August-24 August	170.2(54.3%)

T and P represent mean air temperature and total precipitation amount during the growing season (June-August), respectively.

^a^ The fraction excluded precipitation from the drought plots (in relation to precipitation amount during the growing season) is given in parenthesis.

Irrigation treatment was performed following the drought treatment from 18 July to 1 August in 2013 and from 1 August to 24 August in 2014 (Table 1). The irrigation water was taken from a nearby drinking-water well (mean δ^18^O = -7.72‰) because the isotopic composition of this water was evidently different from that of soil water. In both study years, the irrigation treatments simulated the intensified precipitation events based on the distribution of precipitation events in 2013 and 2014. Hence, two watering events and four watering events were conducted in 2013 and 2014, respectively. For example, on 21 July (200 L) and 31 July (250 L) in 2013, and 12 August (50 L), 15 August (100 L), 18 August (200 L) and 21 August (300 L) in 2014.

### Sampling and environmental monitoring

Each plot included approximately ten *A*. *splendens* tussocks with different kinds of associated species. Three replicates per species (*A*. *splendens* and *A*. *tanguticum*) were randomly collected from each plot (Table 2). Root crown from five individuals per specific grass and herb species were collected on each sampling date such as 5 July, 8 July, 12 July, 18 July, 23 July, 27 July, and 1 August in 2013 and 5 July, 9 July, 14 July, 24 July, 1 August, 15 August, 18 August, 21 August, and 24 August in 2014. The collected root crown were immediately put into glass vials, tightly closed with Teflon-sealed caps, wrapped in Parafilm and kept frozen (-18°C) in the refrigerator prior to the water extraction.

**Table 2:** Information of sampling plants in the control and treatment plots during two consecutive growing seasons.

Species	Family	Life form	Height (cm)	Root depth (cm)
*Achnatherum splendens*	Gramineae	grass	65–100	100–185
*Allium tanguticum*	Liliaceae	herb	15–40	10–48

All the soil samples were collected with a 4-cm-diameter hand-operated bucket auger at 10-cm intervals on each plot. Three replicates per soil samples were collected in each plot during each experimental period. The collected soil samples were used for isotopic and gravimetric soil water content (SWC) analyses. The gravimetric SWC was determined from the weight loss of each sample after they had been dried at 105°C for 24 h.

We measured the predawn (Ψ_p_) and midday (Ψ_m_) leaf water potential of the study plant species in each plot using a WP4 Dewpoint Potential Meter (Wescor, Logan, USA). Predawn measurements occurred from 05:00 to 06:00 h (solar time) and midday measurements occurred during cloudless periods between 12:00 and 14:00 h (solar time). Within each plot, we collected five individuals of each sampling species, and averaged these measurements on each date to obtain a plot-level value for each species.

### Isotopic analyses

Plant tissues and soil samples were extracted using a cryogenic vacuum distillation system [31]. During the extraction process, the extraction time was long enough to fully vaporize the water in both the plant and soil samples. All water isotopic compositions were analyzed using an Isotopic Ratio Infrared Spectroscopy (IRIS) system (Model DLT-100; Los Gatos Research, Mountain View, CA, USA) at the Key Laboratory of Hydropedology, Beijing Normal University (precision of ±1.2‰ for δ^2^H and ±0.3‰ for δ^18^O). The isotopic compositions of the water from the plants were assessed using Spectral Contamination Identifier (LWIA-SCI) post-processing software (Los Gatos Research, Inc.). The stable water isotopic composition was expressed using Eq 1:

$$\delta X=(R_{sample}/R_{standard}-1)*1000,$$ (1)

where *R_sample_* and *R_standard_* represented the sample of the molar abundance ratios (^18^O/^16^O, ^2^H/^1^H) of the sample and the standard (Vienna Standard Mean Ocean Water), respectively.

### Determining the sources of plant water

The difference of isotopic values of water from the plant tissues and its potential sources were directly compared and suggests that water sources have similar isotopic values to stem water represents the water sources extracted by plants [32], but also by using the IsoSource mixing model to estimate the relative proportion of plant water taken up from each source [33]. The source increment and mass balance tolerance were set at 1% and 0.1%, respectively. The distribution (e.g., maximum and minimum values) of feasible solutions and mean absorption proportions on each sampling date was also recorded.

In this study, the water source of a plant was assessed using the δ^18^O calculation in the alpine grassland community. On basis of the soil profiles of SWC and δ^18^O of the soil water, three potential soil water sources were determined: shallow (0–10 cm), middle (10–30 cm), and deep (30–60 cm) soil layers.

### Data analysis

Independent-samples t-test comparisons for the Ψ_p_ and Ψ_m_ between *A*. *splendens* and *A*. *tanguticum*, δ^18^O in soil water, and SWC between control and treatment was used to examine the statistical significance at p<0.05 level. Pearson’s correlations were used to test whether the leaf water potential correlate with soil water content from shallow, middle, and deep soil layers (p<0.05). All the statistical analyses were performed using SPSS 13.0 (SPSS Inc., Chicago, IL, USA).

## Results

### Variations in SWC at the control and treatment plots

**Fig 2:**
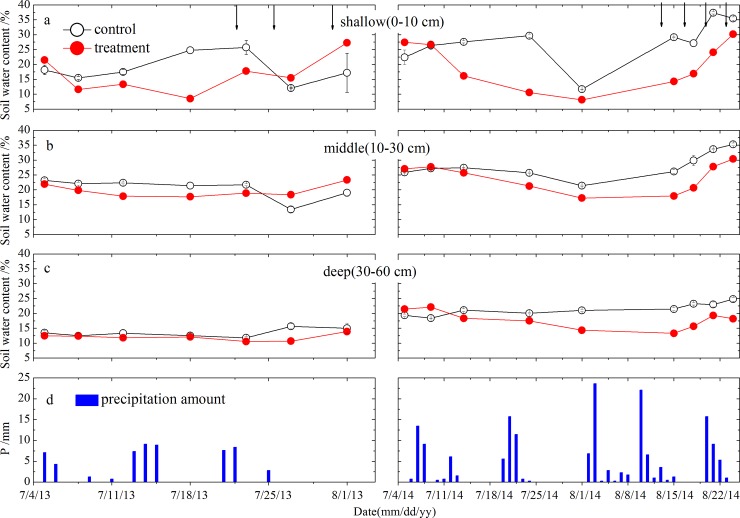
Soil water content (SWC) at the control and treatment plots from shallow soil layer (0-10cm) to deep soil layer (30-60cm). Daily precipitation amount are showed as blue bars in the lowest panels during the study period. The downward arrows indicate the irrigation events. Vertical bar represent standard error.

In both the control and treatment plots, shallow SWC showed greater fluctuations compared with the middle and deep SWC owing to direct water input (e.g., precipitation and irrigation) and evaporation effects (Fig 2). The difference of shallow SWC was found between 2013 and 2014 related with the amount of precipitation events during the studied periods (Fig 2D). There were significant differences in shallow SWC between control and treatment plots (t-test, p<0.05) but become less in middle and deep SWC (t-test, p>0.05, Fig 2). Compared with the control plots, SWC in the shallow soil layer (0–10 cm) was rapidly reduced by the drought treatment, but increased under the different irrigation gradients in each study year (Fig 2A).

In contrast to the shallow soil layer (0–10 cm), the effects of the drought and irrigation treatments on the middle and deep SWC become less pronounced. Middle and deep SWC varied slightly in 2013, whereas they both declined slightly in response to the drought treatments and increased in response to precipitation input and the irrigation treatments in 2014 (Fig 2B and 2C). As with the shallow SWC, the middle and deep SWC in the control plots were slightly higher relative to the levels in the drought plots (Fig 2B and 2C).

### Leaf water potential

**Fig 3:**
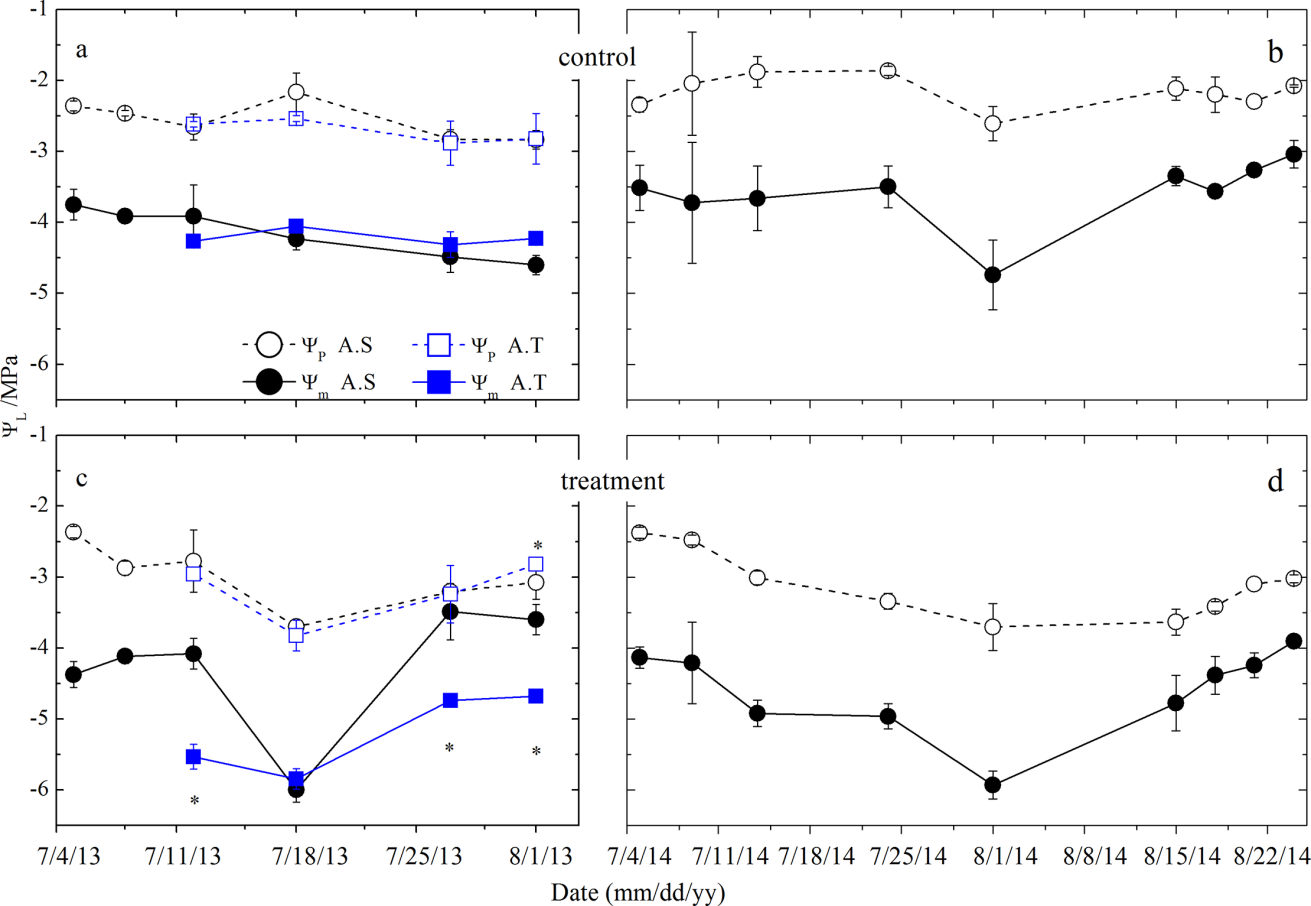
Temporal variations of predawn (Ψ_p_) and midday (Ψ_m_) leaf water potential for plants at the control and treatment plots. *A*.*S* and *A*.*T* represent *A*. *splendens* and *Allium tanguticum*, respectively. Asterisks (*) located above Ψ_p_ or below Ψ_m_ comparisons on each date indicate significant differences between *A*.*S* and *A*.*T* using t-test comparisons at p<0.05.

During the drought treatment, the plant species exhibited significantly low Ψ_p_ and Ψ_m_ compared with the species from the control plots [–3.04±0.16 MPa (drought) versus –2.32±0.08 MPa (control) for Ψ_p_ and –4.92±0.24 MPa (drought) versus –3.93±0.09 MPa (control) for Ψ_m_, t-test, p<0.05], which was similar to the variations in Ψ_p_ and Ψ_m_ in response to the irrigation treatments [–3.25±0.07 MPa (irrigation) versus –2.51±0.10 MPa (control) for Ψ_p_ and –4.26±0.21 MPa (irrigation) versus *–*3.96±0.19 MPa (control) for Ψ_m_, t-test, p<0.05, Fig 3]. Especially for *A*. *splendens*, its Ψ_p_ and Ψ_m_ responded to changes in SWC following both the precipitation event and irrigation treatments in 2013 and 2014.

The leaf Ψ in each species showed temporal variations because of changes in soil water availability (Fig 3). *A*. *splendens* Ψ_p_ varied temporally with the fluctuating SWC, while its Ψ_m_ declined in the control plots during 2013, as seen for its Ψ_p_ and Ψ_m_ during 2014 (Fig 3A and 3B). Ψ_p_ and Ψ_m_ of *A*. *tanguticum* decreased slightly in the control plots. In contrast to the control plots, the effect of drought and irrigation treatment on the Ψ_p_ and Ψ_m_ of each species varied across sampling dates and species, and Ψ_m_ differed significantly between *A*. *splendens* and *A*. *tanguticum* except for the date of 18 July 2013 (t-test, p<0.05, Fig 3C). Mean Ψ_p_ and Ψ_m_ for *A*. *splendens* declined as a result of rainout treatment, but gradually increased due to irrigation (Fig 3C and 3D), similar to that seen for *A*. *tanguticum* (Fig 3C).

Changes in shallow and middle SWC directly influenced Ψ in all species (Table 3). The relationships between Ψ_p_, Ψ_m_, and SWC differed among the soil layers, in that the Ψ_p_ and Ψ_m_ of all species exhibited a significantly positive relationship with shallow and middle SWC (p<0.05).

**Table 3:** Correlation of predawn and midday leaf water potential with soil water content from shallow, middle, and deep soil layers at the control and treatment plots.

Species	Layers	Control		Treatment		No.
		Predawn	Midday	Predawn	Midday	
A.S	Shallow(0–10)	0.756	0.841	0.619	0.652	144
	Middle(10–30)	0.675	0.852	0.539	0.393	144
	Deep(30–60)	0.488	0.561	0.377	0.052	144
A.T	Shallow(0–10)	0.869	0.964	0.784	0.819	36
	Middle(10–30)	0.858	0.563	0.620	0.677	36
	Deep(30–60)	-0.999	-0.771	0.390	0.161	36

### Isotopic variations (δ^18^O) in plant-water sources and plant xylem water

**Fig 4:**
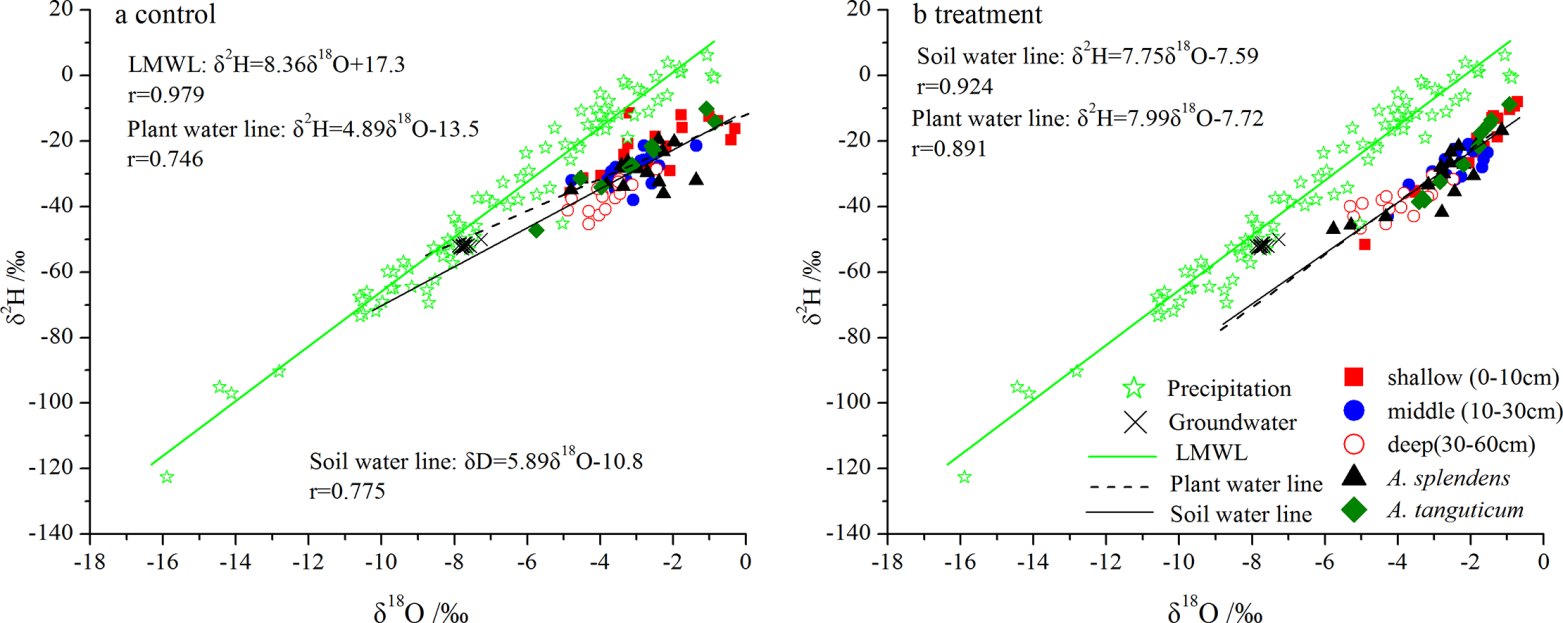
The linear regression relationship between δ^2^H and δ^18^O from soil, plant xylem, ground water and precipitation at the control (a) and treatment (b) plot shown with local meteoric water line (LMWL), soil water line, and plant water line.

The deuterium and oxygen-18 of water from soil and plant xylem were located below the right of local meteoric water line (LMWL, δ^2^H = 8.36δ^18^O+17.3, r = 0.979, Fig 4). The isotopic contents of the groundwater are clustered with LMWL. The slopes of water from soil (5.89) and plant xylem (4.89) at the control plot were lower than those of soil water (7.75) and plant xylem (7.99) at the treatment plot (Fig 4), implying that the isotopic compositions of soil water at the treatment plot were influenced by the irrigation water with more low isotopic values.

**Fig 5:**
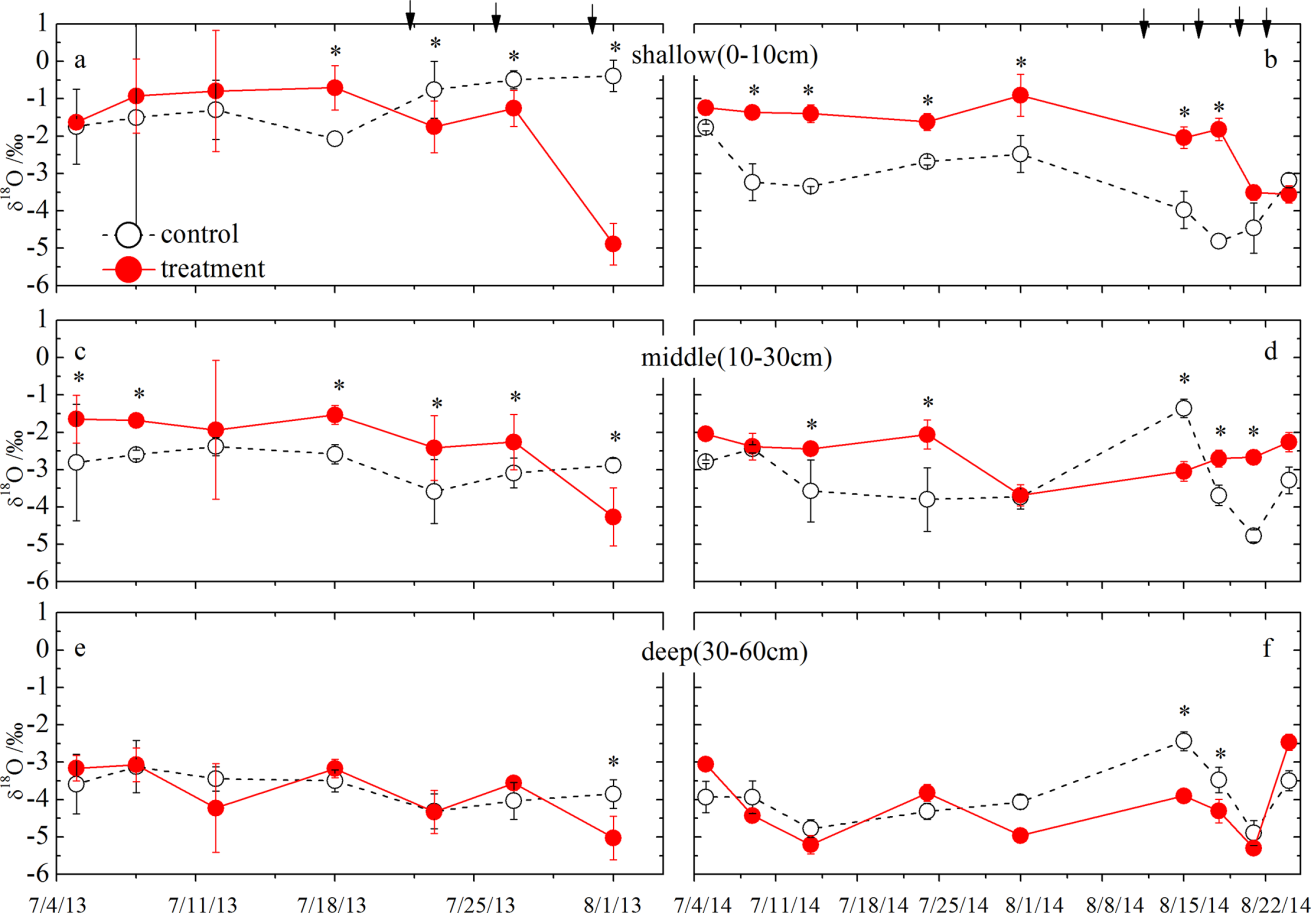
Oxygen-18 content of soil water from the shallow soil layer (0-10cm) to deep soil layer (30-60cm) at the control and treatment plots. The vertical dashed lines separate the drought and irrigation periods. Asterisks (*) indicate significant differences of δ^18^O from shallow, middle and deep soil water between the control and treatment plots using t-test comparisons at p<0.05. The downward arrows indicate the irrigation events.

Soil water δ^18^O from the shallow soil layer (0–10 cm) was highly variable across the sampling dates, suggesting that soil water δ^18^O was affected by source water (e.g., precipitation or irrigation water) with a depleted isotopic composition or evaporative enrichment (Fig 5A). During the drought treatment periods, the difference in shallow soil water δ^18^O between the control and treatment plots (from 5 July to 18 July, 2013) was less pronounced than in 2014 (from 5 July to 1 August) (Fig 5B). The mean shallow soil water δ^18^O (–1.18‰±0.12) was more positive in drought plots than that in control plots (–2.07‰±0.33) during the studied periods.

In contrast to the drought treatment periods, the shallow soil water δ^18^O became more negative with the increased irrigation volume (Fig 5). Similar to the control plots, shallow soil water δ^18^O also declined as a result of precipitation event input, resulting in negative δ^18^O values from 15 August to 24 August in 2014 (Fig 5B, 5D and 5F), which was similarly found for middle soil water. The mean shallow soil water δ^18^O (–2.69‰±0.49) at the drought plots was similar to that at the control plots (–2.82‰±0.67) during the studied periods. In terms of the deep soil layer (30–60 cm) in 2014, its soil water δ^18^O also changed with the high amount of precipitation and larger irrigation volume (Fig 5F).

**Fig 6:**
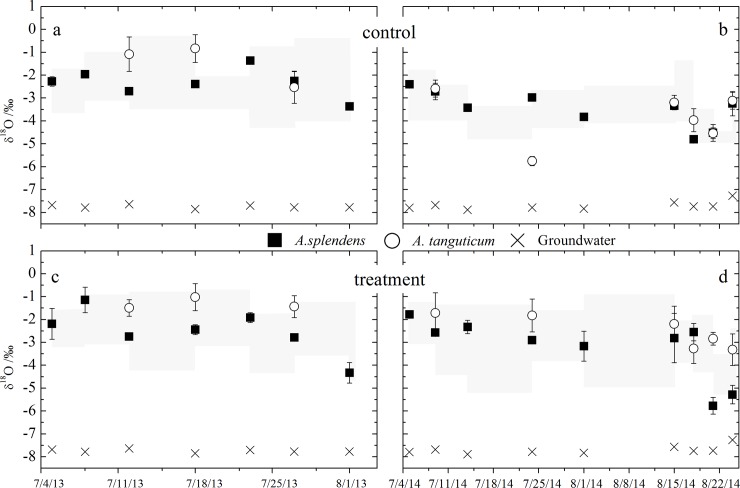
Stable oxygen-18 content of plants tissue for the control (ab) and treatment plots (cd). The left and right columns represent sampling campaign in 2013 and 2014, respectively. Gray filled areas represent the range of mean soil water δ^18^O values in top 60 cm for all plots. Date: mm/dd.

*A*. *splendens* had more depleted isotope signatures (-2.97‰±0.23) relative to *A*. *tanguticum* (-2.43‰±0.52) in the control plots (Fig 6A and 6B). However, plant tissue δ^18^O was more enriched during the drought periods (e.g., –2.36‰±0.20 for *A*. *splendens* and –1.49‰±0.12 for *A*. *tanguticum*) compared with during the irrigation period at the treatment plots (e.g., –3.64‰±0.56 for *A*. *splendens* and –2.59‰±0.23 for *A*. *tanguticum*, Fig 6C and 6D). Moreover, the δ^18^O signatures of most of the plant tissues fell in the range of the soil water δ^18^O signature on all but a few sampling dates (e.g., 18 July, 2013 and 25 July, 2014), indicating that plants were exploiting different water sources from different soil layers at both the control and study plots (Fig 6). The groundwater δ^18^O signature exhibited relatively stable variation (mean −7.72‰±0.15) and differed from soil water and plant tissues at both the control and study plots (Fig 6).

### Determination of plant-water sources

**Fig 7:**
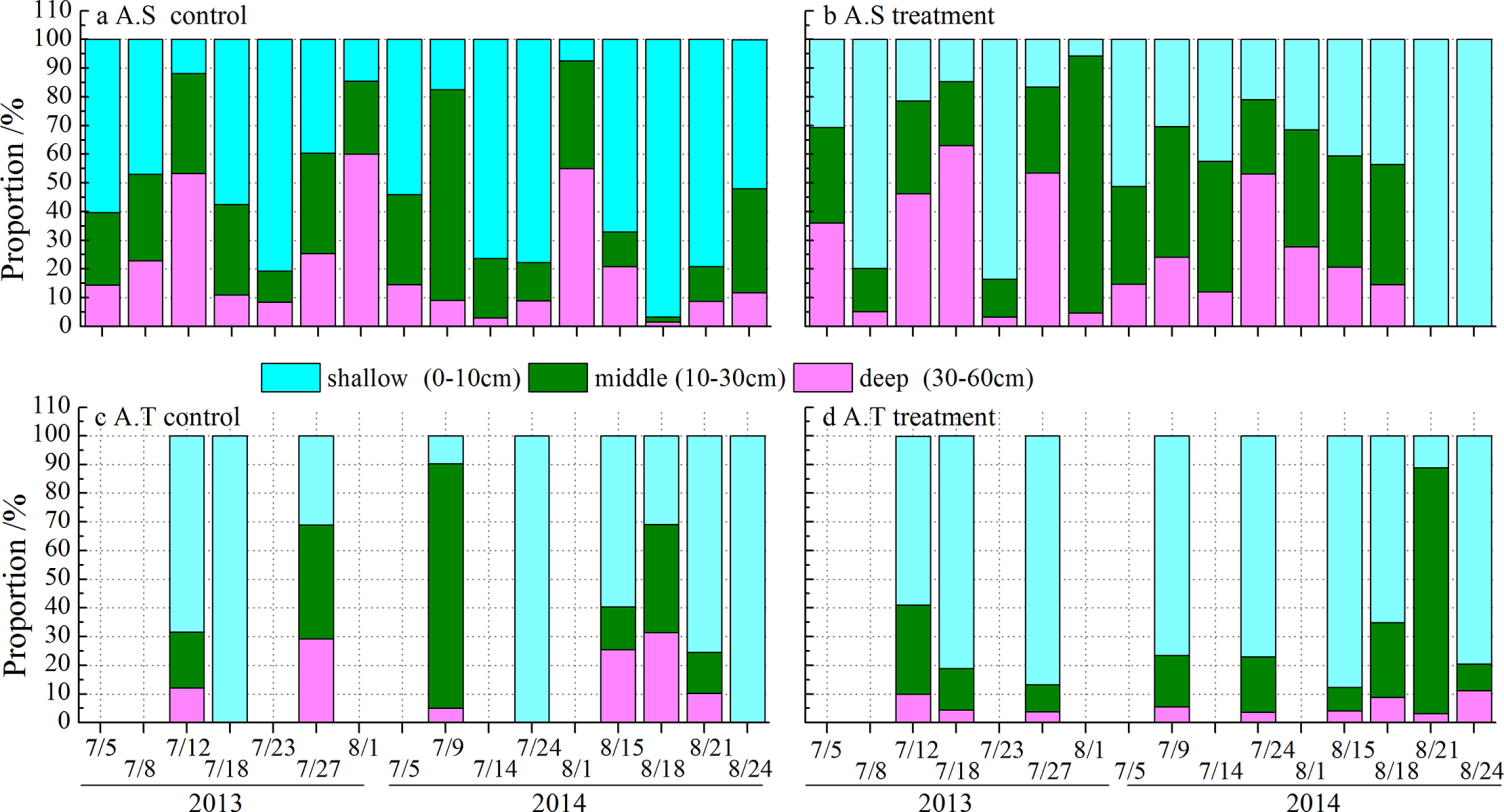
Mean contributions of plant water sources by *A*. *splendens* (ab), and *A*. *tanguticum* (cd) at the treatment and control plots via mixing IsoSource model.

The proportion calculated by the Isosource mixing model revealed that the dominant species in the alpine grassland could extract water simultaneously from the three well-defined water sources: shallow (0–10 cm), middle (10–30 cm) and deep (30–60 cm) soil layers (Fig 7). At the outset of treatment in both 2013 and 2014, *A*. *splendens* mainly absorbed water from the shallow soil layer in both the control (60.4%) and treatment plots (30.8%). Subsequently, as the rainout period progressed, the water source used by *A*. *splendens* gradually shifted from the shallow soil layer to the deep soil layer as a result of the decline in shallow SWC in the treatment plots (e.g., from 8 July to 18 July, 2013 and 9 July to 1 August, 2014). However, under artificial irrigation treatments, the shallow soil layer contributed the most to water absorption for *A*. *splendens*, especially in 2014, when this species obtained all its water from the shallow soil layer (e.g., 21 and 24 August 2014) as a result of high shallow SWC.

In contrast to the treatment plots, the contributions of the three soil layers to total plant water uptake in the control plots were more variable over time because of changes in SWC (Fig 7). The contribution of water from the deep soil layer to uptake by *A*. *splendens* increased from 14.4% to 53.3% between 5 July and 12 July, 2013 (Fig 7A), and this change was also observed in 2014 as a result of the number of consecutive rainless days before each sampling date (2.9% on 14 July, 2014 to 55% on 1 August, 2014, Fig 7B). The effect of precipitation input on the control plots was obvious in the shallow SWC, and, thus, *A*. *splendens* mostly depended on water from this soil layer (52%–96.7%), particularly during August 2014 following substantial precipitation events (Fig 7A). Unlike the water patterns in *A*. *splendens*, and *A*. *tanguticum* extracted a higher proportion of water from the shallow and middle soil layers (31%–100%), and their water-use patterns did not obviously change in either the treatment or control plots (Fig 7).

## Discussion

### Plant water-relation response to soil water availability

Natural seasonal and experimentally altered changes in SWC significantly influenced the water relations and significantly differed among the studied species, indicating that these species responded different to the levels of precipitation during the growing season [1, 17, 21, 34]. Meanwhile, their Ψ was largely determined by shallow SWC as indicate by the significant positive relationship between these two factors, suggesting that Ψ is a function of shallow SWC [18, 35, 36]. In this study, *A*. *splendens* Ψ_p_ and Ψ_m_ increased after precipitation and irrigation water input in 2014; however, when little rainfall fell during late July, 2013, Ψ in this species steadily declined with the decrease in shallow SWC and significantly correlated with shallow SWC (Table 3). Hence, *A*. *splendens* water status (Ψ) improved in response to the rain-recharged shallow soil water, which could be closely associated with its flexible water-use pattern among the multiple soil layers, as reported previously by Kray et al. (2012) [36]. These authors observed a similar Ψ response to a rain-driven increase in surface soil moisture for *Sarcobatus vermiculatus* in southern Colorado following unusual levels of summer precipitation, and showed that the plant was also able to acquire water from deep soil layers (e.g., groundwater).

Similar to *A*. *splendens*, shallow-rooted *A*. *tanguticum* had significant Ψ responses to the natural seasonal and experimentally controlled soil water availability in 2013, suggesting that both species obtained water via roots that were concentrated in the shallow soil layer. Williams and Ehleringer (2000) [35] also found plant water status (Ψ) to be closely correlated with shallow soil water availability, which is further supported by the significant relationships between shallow SWC and Ψ (Table 3). Another study in California [37] similarly reported that Ψ_p_ in the shallow-rooted grass *Distichlis spicata* was also responsive to shallow soil water availability, despite the presence of a shallow water table. In the irrigation treatment plots in particular, irrigation water resulted in a synchronous increase in Ψ_p_ and Ψ_m_ for *A*. *splendens* and *A*. *tanguticum* in 2013, implying that both species were able to recover overnight from very low daytime Ψ, as also observed in *Stipa tenacissima* grassland [38]; the authors of the latter study suggested that grasses use summer rain pulses to improve their growth and physiological performance. Although the rainout treatment led to significantly low Ψ for the studied species, their Ψ gradually increased in response to the improvement in shallow SWC following artificial irrigation in 2013 and 2014. Therefore, the effect of shallow soil moisture on the improvement in Ψ for shallow-rooted species is significant and may affect their growth during the growing seasons.

### Water sources of the study plants

The distinct δ^18^O signature of water from the plant tissues and potential water sources (i.e., groundwater and soil water) enabled us to distinguish the different sources of water used by the study species [39, 40]. In this study, seasonal and specific-species differences in δ^18^O content were observed in both the control and treatment plots (Figs 5 and 6), indicating that plants acquired water from diverse water pools in space and time to support transpiration during the growing season. These results were consistent with those of previous studies in other semiarid communities [13, 41, 42]. Furthermore, at both the control and treatment plots, *A*. *splendens* and *A*. *tanguticum* totally independent from the groundwater because of great difference of water δ^18^O values between plant tissue and groundwater (Fig 6). Recent published studies also demonstrated that grasses are more inclined to exploit soil water pools rather than groundwater because of their limited root distribution and nutrient availability[17, 36, 43]; such a pattern results in more carbon storage in the shallow soil layer to capture the summer rain pulse for grasses in semiarid regions [9, 44]. It can be clearly observed that the proportion of irrigation water (simulated rainfall pulse) utilized by plants tended to increase with the volume of water added (see S1 Table).

The seasonal patterns in water-use sources varied greatly across the studied species. The differences observed in the use of water sources between the drought and control treatments could be a short-term response to soil water availability. The depth of water absorption by *A*. *splendens* was significantly affected by drought and artificial irrigation, and this species tended to acquire water from the deep soil layer during the drought periods (Fig 7). Furthermore, *A*. *splendens* in the control plots was also observed to shift its water source to the deep soil layers in response to the prolonged run of rainless days in both 2013 and 2014 (Fig 7). These results are in agreement with a previous study on the Colorado Plateau by Schwinning *et al*. (2005) [13], who demonstrated that the depth of water-use source by woody (*Gutierrezia sarothrae* and *Ceratoides lanata*) and grass (*Oryzopsis hymenoides*) species changed between shallow and deeper soil water in response to soil water conditions. Hoekstra *et al*. (2014) [21] also reported that short-term experimental drought could result in a shift in water uptake from the deep soil layers in both shallow- and deep-rooted species, closely linked with the dynamic variations in belowground root biomass among soil layers in response to drought [2, 19]. Thus, this shift between deep soil layers and shallow soil layers as the major water source appears to be important for species growing in water-limited environments, especially alpine regions [45, 46].

In contrast to *A*. *splendens*, *A*. *tanguticum* both generally utilized a single water source, depending on water from the shallow soil layer, with no shift to water uptake from the deep soil layer in response to drought (Fig 7). These results are supported by numerous recent studies reporting shallow rooting patterns and water uptake of grassland species affected by drought. For instance, Nippert et al. (2007) [16] revealed that C_4_-grasses had a greater dependency on water from the top soil layer (0–30 cm) than from the deep soil layer (>30 cm). This dependency on shallow soil water even continued during prolonged dry periods because of most active surface roots gathering in the shallow soil layers [35, 39], which is in accordance with the results from the current study. A recently published tracer (δ^2^H) study also reported that grasses in a mesic savanna continued to obtain water from a shallow soil depth (0–5 cm) even when water became scarce [23]. Overall, our data from alpine grasslands conform well to previously published works on grasslands, which have evolved under more arid climates, and suggest that this kind of shallow-rooted species (e.g., *A*. *tanguticum*) mainly depend on water from the shallow soil layers rather than shifting to deeper layers under drought conditions. In addition, a study of *L*. *chinensis* in Inner Mongolia (China) by Yang et al. (2010) [10] reported a resource-dependent water-use strategy that enabled this species to adjust its uptake depth according to the soil water availability, similar to that found for *A*. *splendens*; and this might be closely associated with soil texture and root architecture. However, this was not distinguished in our results. The uncertainty on water uptake by *A*. *tanguticum*) may be related with its efficient water extraction or stomatal control and these should be studied further.

Differences in functional rooting depth and physiological traits for the plant species we studied could explain the difference in their water-use patterns in response to soil water availability. *A*. *splendens* showed flexibility in its water-use source and alleviated water stress resulting from transpiration with high Ψ. By contrast, *A*. *tanguticum* were more susceptible to water stress, with low Ψ, and might use osmotic adjustments to maintain their xylem conductivity and leaf cell turgor, which would enable these species to transpire and extract water from increasingly dry shallow soils [47, 48]. These traits indicate that coexisting grass species have different adaptive patterns to avoid direct competition for water in alpine grasslands, and are widespread among different climate regimes that promote drought resilience [49]. The lower Ψ_m_ (–5.84 MPa) measured within the rainout plots in 2013 likely reflect osmotic adjustments by *A*. *tanguticum* that enable this species to acquire water from the shallow soil layer, as also observed by Prechsl *et al*. (2015) [17], who suggested that plants are still able to extract water at a relatively low SWC, as confirmed by Ψ and gas exchange measurements. Thus, physiological acclimation and root functional traits appear to facilitate the uptake of enough water for transpiration under drought, improving the ecological fitness of grasses under varying environmental conditions.

### Wider implications

In the study region, the precipitation patterns have altered significantly in recent decades, with an increasing trend in the number of both small and large precipitation events, the longest run of consecutive rainless days, and the total number of days with more than ten consecutive rainless days from the 1960s to the 1990s [25]. Thus, we predict that, if frequent and small events or occasional and large events occur at the peak of the growing season (as simulated by irrigation treatment in the current study), the water relations of the studied species would improve [2]. Reduced water stress could increase the productivity and cover of these species and further enhance their above- and belowground competition, such as for water or nutrients, as a result of the higher canopy of *A*. *splendens* compared with the other two species. This pattern would increase the evapotranspiration from the shallow soil layer, gradually using up this water supply, thus facilitating the growth of *A*. *splendens*, given that it has a more flexible rooting strategy that allows greater access to deeper soil water. If long runs of consecutive rainless days were to occur occasionally (as simulated by the drought treatment in the current study), the shallow soil moisture would be reduced, which might enhance the water stress of *A*. *tanguticum* compared with *A*. *splendens*. Thus, the precipitation patterns could affect the water-use patterns of coexisting plants, which might have further implications for the distribution of grass species in alpine and semiarid grasslands. Hence, from a long-term perspective, the present research demonstrated that the flexible patterns in water use by *A*. *splendens* could explain its dominance across a broad range of alpine conditions around Qinghai Lake, China, in response to recent climate changes.

## Conclusions

Based on our results from the alpine grassland community around Qinghai Lake, there was evidence that *A*. *splendens* could change its water source from shallow (0–10 cm) to deep soil layers (30–60 cm) in response to seasonal soil water availability, whereas shallow-rooted *A*. *tanguticum* largely depended on water from shallow soil layers. By contrast, the water relations of all the species studied were sensitive to changes in shallow SWC, indicating that the extent of shallow SWC has a vital role in their coexistence during, and acclimation to, variable summer precipitation. Overall, alpine grass species adjusted not only their water-use patterns to adapt to a semi-arid environment, but also their physiological traits (e.g., Ψ). Hence, changes in growing season precipitation are most likely to affect the shallow-rooted *A*. *tanguticum*, but less so *A*. *splendens*. Persistent changes in precipitation patterns could cause a shift in the plant community composition of this alpine grassland, enabling those species with flexible water-use strategies to dominate the habitats around Qinghai Lake under future climate scenarios.

## Supporting information

S1 Fig Photo of drought treatment. (DOCX)

S1 Table Proportional use of irrigation water by plant species as determined by the two-end-member equation. (DOCX)
