# Supplementary figures and images for: Contrasting response of coexisting plant’s water-use patterns to experimental precipitation manipulation in an alpine grassland community of Qinghai Lake watershed, China
Source: PLoS One. 2018 Apr 20;13(4):e0194242. doi: 10.1371/journal.pone.0194242 (PMC5909899; doi:10.1371/journal.pone.0194242)

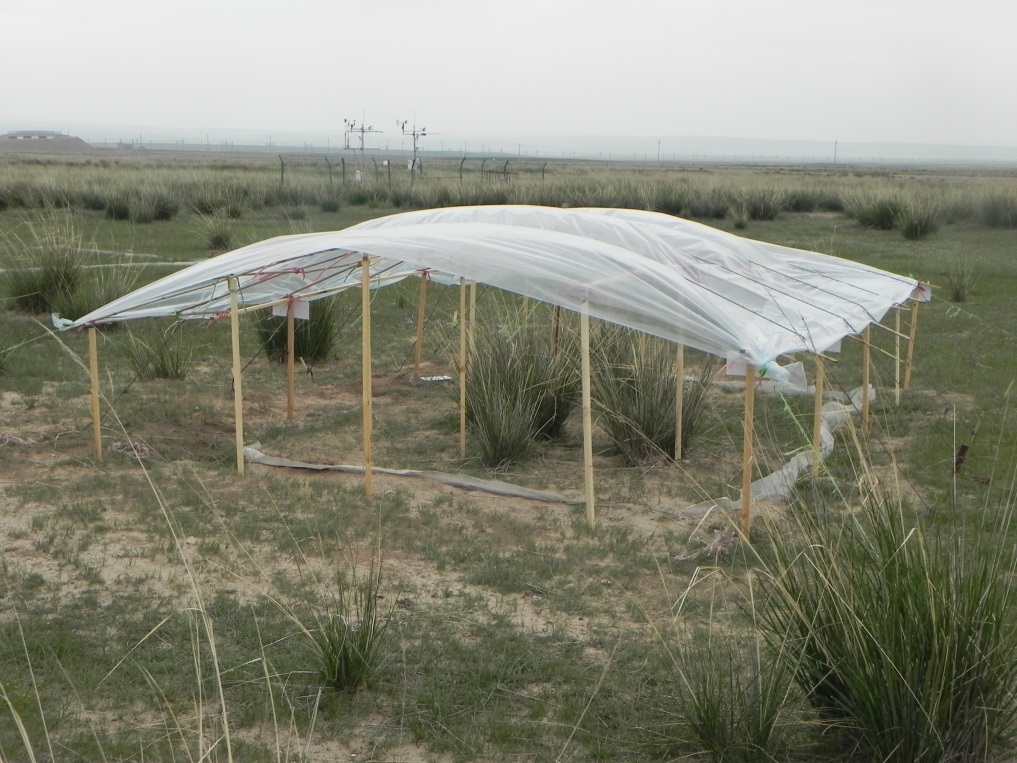


Figure S1 Photo of drought treatment.

Supplement: S1 Fig — (DOCX) [file pone.0194242.s001.docx]
