# Supplementary material for: Contrasting response of coexisting plant’s water-use patterns to experimental precipitation manipulation in an alpine grassland community of Qinghai Lake watershed, China
Source: PLoS One. 2018 Apr 20;13(4):e0194242. doi: 10.1371/journal.pone.0194242 (PMC5909899; doi:10.1371/journal.pone.0194242)
Supplement: S1 Table — (DOCX) [file pone.0194242.s002.docx]

Table S1 Proportion use of irrigation water by plant species via the two-end-member equation

| Date | *A. splendens* | | *A. tanguticum* | |
| --- | --- | --- | --- | --- |
|  | shallow | irrigation | shallow | irrigation |
| 2013/7/23 | 0.97 | 0.03 | ns | ns |
| 2013/7/27 | 0.76 | 0.24 | 0.97 | 0.03 |
| 2013/8/1 | - | - | - | - |
| 2014/8/15 | 0.86 | 0.14 | 0.97 | 0.03 |
| 2014/8/18 | 0.87 | 0.13 | 0.75 | 0.25 |
| 2014/8/21 | 0.33 | 0.67 | 0.83 | 0.17 |
| 2014/8/24 | 0.59 | 0.41 | 0.85 | 0.15 |

- represents impossible to calculate by the two-end-member mixing equation because of the plant tissue δ^18^O excluding the range of δ^18^O from shallow soil water and irrigation water. ns represents no sampling for corresponding plant during the study period.

A two-end-member mixing equation ([Dawson, 1993](#_ENREF_1)) was used to estimate the proportion of irrigation water(groundwater) used by plants we studied. The form of the equation is shown as follows:

$$\delta_{plant}=f_{1}\delta_{s}+f_{2}\delta_{i}$$

$$1=f_{1}+f_{2}$$

Where $\delta_{s}$ and $\delta_{i}$ represent the oxygen-18 content in the shallow soil water and irrigation water, respectively. $\delta_{plant}$ represents the specific-species oxygen-18 content.

Reference

Dawson TE. 1993. Hydraulic lift and water use by plants: implications for water balance, performance and plant-plant interactions. Oecologia, **95**: 565-574.
